# MR-guided SBRT boost for patients with locally advanced or recurrent gynecological cancers ineligible for brachytherapy: feasibility and early clinical experience

**DOI:** 10.1186/s13014-022-01981-z

**Published:** 2022-01-15

**Authors:** Indrawati Hadi, Chukwuka Eze, Stephan Schönecker, Rieke von Bestenbostel, Paul Rogowski, Lukas Nierer, Raphael Bodensohn, Michael Reiner, Guillaume Landry, Claus Belka, Maximilian Niyazi, Stefanie Corradini

**Affiliations:** 1 Department of Radiation Oncology, University Hospital, LMU Munich, Marchioninistrasse 15, 81377 Munich, Germany; 2 German Cancer Consortium (DKTK), Partner site Munich, Munich, Germany

**Keywords:** Cervical cancer, Vaginal cancer, Recurrent, Gynecological cancer, Stereotactic, SBRT, Brachytherapy, Mr-guided radiotherapy

## Abstract

**Background and purpose:**

Chemoradiotherapy (CRT) followed by a brachytherapy (BT) boost is the standard of care for patients with locally advanced or recurrent gynecological cancer (LARGC). However, not every patient is suitable for BT. Therefore, we investigated the feasibility of an MR-guided SBRT boost (MRg-SBRT boost) following CRT of the pelvis.

**Material and methods:**

Ten patients with LARGC were analyzed retrospectively. The patients were not suitable for BT due to extensive infiltration of the pelvic wall (10%), other adjacent organs (30%), or both (50%), or ineligibility for anesthesia (10%). Online-adaptive treatment planning was performed to control for interfractional anatomical changes. Treatment parameters and toxicity were evaluated to assess the feasibility of MRg-SBRT boost.

**Results:**

MRg-SBRT boost was delivered to a median total dose of 21.0 Gy in 4 fractions. The median optimized PTV (PTV_opt_) size was 43.5ccm. The median cumulative dose of 73.6Gy_10_ was delivered to PTV_opt_. The cumulative median D2ccm of the rectum was 63.7 Gy; bladder 72.2 Gy; sigmoid 65.8 Gy; bowel 59.9 Gy (EQD2_3_). The median overall treatment time/fraction was 77 min, including the adaptive workflow in 100% of fractions. The median duration of the entire treatment was 50 days. After a median follow-up of 9 months, we observed no CTCAE ≥ °II toxicities.

**Conclusion:**

These early results report the feasibility of an MRg-SBRT boost approach in patients with LARGC, who were not candidates for BT. When classical BT-OAR constraints are followed, the therapy was well tolerated. Long-term follow-up is needed to validate the results.

## Background

External beam radiotherapy (EBRT) with concurrent chemotherapy followed by a brachytherapy (BT) boost is the standard of care for most patients with locally advanced gynecological cancer [1]. On the other hand, the management of patients with non-metastasized recurrent gynecological cancer remains a challenge. If chemoradiotherapy followed by BT boost has not been performed before, this could be a treatment option to prevent extensive surgical resection, such as pelvic exenteration.

Even though the utilization of a sequential BT boost improves the outcomes in patients with locally advanced or recurrent gynecological cancer (LARGC) [2–4], not every patient is suitable for a BT boost due to the extent or localization of the tumor, bone infiltration, infiltration of the pelvic wall or adjacent organs, or in multimorbid patients, who cannot undergo anesthesia.

As an alternative, the application of a stereotactic body radiotherapy (SBRT) boost delivered with cone beam computed tomography (CBCT) as image guidance and a linear accelerator (LINAC) has been performed in previous studies [5, 6]. However, due to the reduced soft tissue contrast, it remains challenging for the radiation oncologist to differentiate between tumor and normal tissue based on CBCT. As it is important to deliver high doses to the high-risk clinical target volume (HR-CTV) while sparing critical organs at risk (OAR), the use of high precision radiotherapy becomes indispensable.

Magnet resonance imaging (MRI) provides superior soft tissue contrast compared to CBCT. MRI-guided linear accelerators (MR-Linac) have incorporated an MRI scanner into a radiation therapy delivery system, allowing an improved visualization of gynecologic tumors and OAR [7, 8]. Online MR-guided radiotherapy (MRgRT) also provides the opportunity to adapt the treatment plan to interfractional anatomical changes and monitor intrafractional motion [9–11], making this method a considerable option for patients with LARGC, who are not eligible for BT. In the present study, we investigated the feasibility and safety of an MRg-SBRT boost following EBRT.

## Methods

### Patients

Ten consecutive patients with non-metastatic LARGC and ineligibility for BT were treated between 03/2020 and 03/2021. The median age at the beginning of treatment was 56 years (range, 33–82 years). Five patients (50%) had recurrent cervical cancer, 2 patients (20%) had a locally advanced cervical cancer, and 3 patients (30%) had locally advanced recurrent vaginal cancer.

**Fig. 1:**
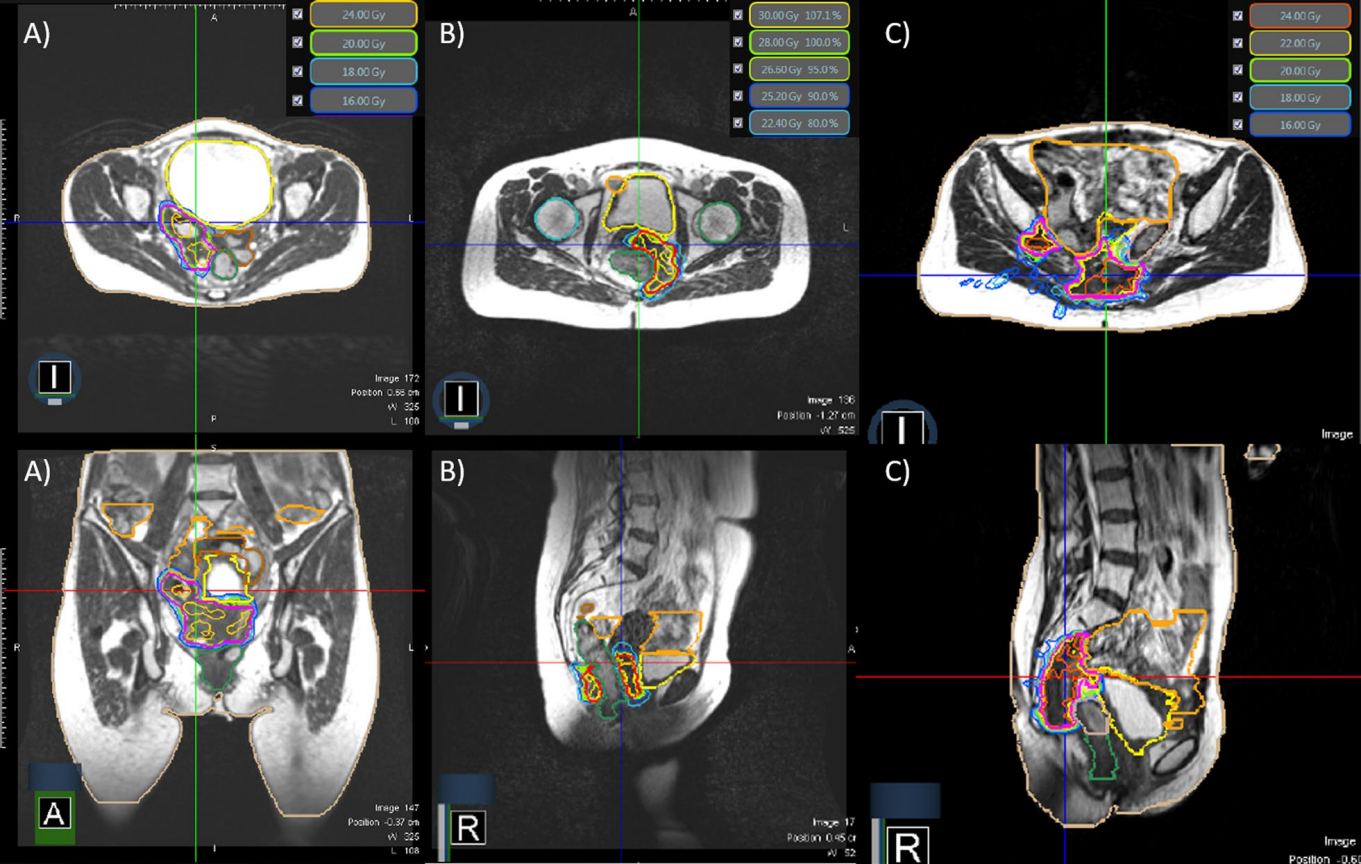
**A–C** **A** 33 y/o patient with recurrent cervical cancer extending from the recto-sigmoidal junction to the upper third of the vagina with infiltration of the uterus, the right ovary and the rectum. She received a MR-guided SBRT boost to the PTV_opt_ (131.35 ccm) with a single dose of 5 Gy and total dose of 20 Gy q.a.d (total EQD2 = 69.3Gy_10_). **B** 57 y/o patient with the diagnosis of cT4 cervical cancer with infiltration of the rectum and the pelvic floor. She was treated with a MRg-SBRT boost of 28 Gy in 4 fraction dose q.a.d to the PTV_opt_ (89.97 ccm) resulting in a cumulative EQD2 = 83.9Gy_10_. **C** 47 y/o patients with recurrent cervical cancer that extended to the presacral region, through the sciatic foramen, with erosion of the ileum bone and infiltration of sciatic nerve. The MRg-SBRT boost was performed with 20 Gy à 5 Gy q.a.d to the PTV_opt_ (126.73 ccm), total EQD2 = 75.0Gy_10_

BT eligibility was assessed for all patients by an experienced brachytherapist specialized in gynecological tumors. In the setting of recurrent disease, a biopsy was obtained before the treatment. The decisions for the treatment approach was made by an interdisciplinary tumor board. Patients underwent MRI and ^18^FDG-PET/CT and gave informed consent prior to therapy. The patients were not suitable for BT due to: extensive infiltration of the pelvic wall alone in 10%, extensive infiltration of other organs/structures (urethra, rectum, sigma, ureter, pelvic floor, and bladder) in 30%, and both infiltration of the pelvic wall and adjacent organs in 50% (Fig. 1). The BT boost could not be performed in one patient (10%) due to ineligibility for anesthesia and multiple comorbidities. Patient characteristics are summarized in Table 1. This retrospective analysis was approved by ethic committee of the LMU Munich on record number EK 20–291.

**Table 1:** Patients characteristics

No	Age	Diagnosis	Reason for BT boost ineligibility	Outcome after ≥ 3 months after therapy
1	33	Recurrent cervical cancer	Infiltration of the pelvic wall. Tumor extended from the recto-sigmoidal junction to the upper third of the vagina with infiltration of the uterus, the right ovary and the rectum	PD 6 months after therapy with distant metastasis
**2**	47	Recurrent cervical cancer	Infiltration of pelvic wall, presacral region, through the sciatic foramen. Erosion of the ilium and infiltration of the sciatic nerve	PR
**3**	56	Recurrent cervical cancer	Infiltration of the urinary bladder wall. The tumor circumscribed the left ureter and extended to the sigmoid	CR of local tumor. One lymph node metastasis inguinal right; outside radiation field
**4**	56	Recurrent cervical cancer	Infiltration of the pelvic wall and left ureter	CR
**5**	37	Recurrent cervical cancer	Infiltration of the pelvic floor, ureter, paravaginal region and sigmoid	CR
**6**	70	Recurrent vaginal cancer	Infiltration of the pelvic floor	CR
**7**	43	Recurrent vaginal cancer	Contact with rectosigmoid junction and left ovary	PR
**8**	63	Recurrent vaginal cancer	Infiltration of the urethra	CR
**9**	57	1st diagnosis of cervical cancer	Infiltration of the pelvic floor and rectum (cT4)	CR
**10**	82	1st diagnosis of cervical cancer	not suitable for anesthesia	PD, distant metastases 7 months after therapy

*PD* progressive disease, *PR* partial remission, *CR* complete remission

### Concurrent chemoradiotherapy

EBRT of the pelvic (80%) and paraaortic lymphatics (20%) was applied using a volumetric modulated arc therapy (VMAT) technique and a dose per fraction of 1.8 Gy to a total dose of 45.0 Gy. A simultaneous integrated boost (SIB) to the primary tumor was given in 4 patients with a total dose of 50.0–55.0 Gy in 25 fractions, while 2 patients received a sequential boost to the primary tumor with a total dose of 10.0–16.0 Gy in 2.0 Gy per fraction. Moreover, a SIB to positive lymph nodes was given in 3 patients to a total dose of 55.0–57.5 Gy in 25 fractions. Nine patients (90%) received concurrent chemotherapy with cisplatin 40 mg/m^2^ weekly, with a median of 5 cycles (range: 4–5 cycles), while carboplatin AUC 2–3 weekly was given in 1 patient due to chronic renal insufficiency for 3 cycles. Treatment parameters of chemoradiotherapy are summarized in Table 2.

**Table 2:** Treatment parameters

Treatment parameters	Median (range)
*EBRT*	
Dose of pelvic irradiation	45.0 Gy in 25 fx
SIB to primary tumors (n = 4)	50.0–55.0 Gy in 25 fx
Sequential boost to the primary tumor (n = 2)	10–16 Gy in 2 Gy/fx
Total dose of primary tumor and pelvic/paraaortic lymphatic pathways	45.0 Gy (45.0–55.0 Gy)
SIB to positive lymph nodes (n = 3)	55.0–57.5 Gy
Concurrent chemotherapy with cisplatin 40 mg/m^2^ weekly (n = 9)	5 cycles (4–5 cycles)
Concurrent chemotherapy with carboplatin AUC 2 weekly (n = 1)	3 cycles
*MR-guided SBRT boost*	
Dose per fraction	5.2 Gy (4.0–7.0 Gy)
Total dose	21.0 Gy (8.0–28.0 Gy)
HR-CTV	35.2 ccm (12.1–111.75 ccm)
PTVopt	43.5 ccm (24.2–131.35 ccm)
Net beam on time/fraction	5.6 min (1.53–11.67 min)
Overall treatment time/fraction	77 min (44.0–89.5 min)
Rectum D2ccm (EQD2_3_)	17.6 Gy_3_ (1.5–24.9 Gy_3_)
Bladder D2ccm (EQD2_3_)	25.4 Gy_3_ (5.1–35.2 Gy_3_)
Sigmoid D2ccm (EQD2_3_)	17.4 Gy_3_ (0.3–24.9Gy_3_)
Bowel D2ccm (EQD2_3_)	11.6 Gy_3_ (0.3–23.6 Gy_3_)
*Combined treatment (EBRT + MR-guided SBRT boost)*	
Cumulative total dose (EQD2_10_)	73.6 Gy_**10**_ (69.3–83.9 Gy_10_)
Rectum D2ccm (EQD2_3_)	63.7 Gy_3_ (51.5–72.6 Gy_3_)
Bladder D2ccm (EQD2_3_)	72.2 Gy_3_ (59.7–83.6 Gy_3_)
Sigmoid D2ccm (EQD2_3_)	65.8 Gy_3_ (43.9–69.0 Gy_3_)
Bowel D2ccm (EQD2_3_)	59.9 Gy_3_ (47.7–70.0 Gy_3_)
Overall treatment time (OTT)	50 days (39–56 days)

*Fx* fractions

### MRg-SBRT boost

A diagnostic MRI of the pelvis was performed at the 4th week of EBRT to assess early response to chemoradiotherapy. The SBRT boost was performed using a 0.35 T hybrid MR-Linac (Viewray Inc., Mountain View, CA).

#### Treatment simulation

Simulation of the MRg-SBRT boost was conducted a week before the treatment. All patients were required to empty the rectum (using an enema, if necessary). Scopolamine butylbromide (Buscopan) was given 30 min before the simulation to reduce bowel movements and improve the quality of MR imaging. We administered ultrasound gel to dilate the vagina and allow a better visualization of the vagina and tumor.

Patients were then immobilized in the supine position with the arms parallel to the body or above the head using a dedicated positioning device (MRI Wing step, ITV, Innsbruck, Austria). The MRI surface receive coils were located anteriorly and posteriorly to the patients. The MRI scan was performed using true fast imaging (TRUFI)-sequences in free-breathing (FB). A standard planning CT was acquired subsequently using the same patient positioning to obtain electron density information. Both MRI and CT imaging were co-registered using a deformable registration algorithm.

Target volume and organs at risk (OAR) delineation were adapted from the BT recommendations of the Gynaecological GEC-ESTRO Working Group [12]. In case of primary cervical cancer, the high-risk clinical target volume (HR-CTV) was defined as macroscopic tumor and the remaining cervix [13]. In the recurrent setting, we defined the HR-CTV as macroscopic tumor and adjacent areas considered to contain microscopic spread. The HR-CTV was expanded 5 mm isotropically to generate the planning target volume (PTV). Afterwards, an optimized PTV (PTV_opt_) was obtained from subtracting organs at risk (OAR) with a 3 mm isotropic expansion from the PTV. We applied the currently recommended BT-constraints for OARs [14].

#### Treatment delivery

The aforementioned patient preparations were also required prior to each treatment delivery. An MRI scan was obtained thereafter using TRUFI-sequences. As part of the daily online-adaptive workflow, we re-contoured the target volumes and OARs to adapt for interfractional changes. A new radiation plan was subsequently optimized based on predefined constraints and current anatomical variations.

During the treatment delivery, we monitored target volume and OAR motion using continuous, real-time 2D Cine MRI in a sagittal plane. We defined a gating boundary contour by expanding the GTV 3 mm isotropically. Using a deformable registration-based tracking algorithm, the beam was gated automatically. The maximum percentage of GTV, which is allowed to be outside the boundary region, was 5%. Above this threshold, the system stops the beam automatically [15].

Biologically equivalent doses in 2 Gy per fraction (EQD2) were applied to sum up the total dose from EBRT and the MRg-SBRT boost. EQD2 of normal tissue was calculated with α/β = 3 Gy and EQD2 of tumor was obtained with α/β = 10 Gy.

### Statistical analysis

Patient demographics were calculated using descriptive statistics as absolute and relative frequencies. Treatment parameters and toxicity were evaluated to assess the feasibility of MRg-SBRT boost. Statistical analyses were done with IBM SPSS Statistics, Version 26 (IBM, Armonk, New York, USA).

## Results

### Treatment parameters

The MRg-SBRT boost was delivered every other day (q.a.d). The median of applied fractions was 4 (range: 2–4 fractions). The median dose per fraction was 5.2 Gy (range: 4.0–7.0 Gy) and median total dose was 21.0 Gy (range: 8.0–28.0 Gy). The median HR-CTV volume was 35.2 ccm (range, 12.1–111.75 ccm) and the median size of PTV_opt_ was 43.5 ccm (range: 24.2–131.35 ccm). The cumulative total dose of the combined treatment (EBRT + MR-boost) of the PTV_opt_ was in median 73.6 Gy (range: 69.3–83.9 Gy EQD2_10_). The rectum received a cumulative median D2ccm of 63.7 Gy_3_ (range: 51.5–72.6 Gy_3_) and the median cumulative D2ccm of other OARs were as follows: 72.2Gy_3_ (range: 59.7–83.6Gy_3_) for bladder; 65.8Gy_3_ (range: 43.9–69.9 Gy_3_) for sigmoid; and 59.9 Gy_3_ (range: 47.7–70.0 Gy_3_) for bowel. The median net beam on time/fraction was 5.6 min (range: 1.53–11.67 min), and the median overall treatment time/fraction was 77 min (range: 44.0–89.5 min), including the adaptive workflow which was applied in 100% of fractions. The median duration from the beginning of EBRT to the last SBRT fraction was 50 days (range: 39–56 days). All treatment parameters are summarized in Table 2.

## Outcomes

With a median follow-up of 9 months (range, 8–19 months), a complete response (CR) was observed in 6 patients (60%), while local control was reported for 9 patients (90%). One of the 6 patients with CR developed inguinal lymph node metastases outside the radiation field within 12 months after the end of the MRg-SBRT boost. The patient received a salvage radiation treatment of the right inguinal lymphatic pathways with SIBs to lymph node metastases and is currently with no evidence of disease. Partial remission was seen in 2 patients, while a progression was reported in 2 patients: 1 patient developed distant metastases to mediastinal lymph nodes 7 months after the treatment, without locoregional recurrence; while 1 patient developed a local progression in the pelvis as well as distant metastasis within 6 months after therapy. These two patients were treated with salvage systemic therapy. Patient outcomes are described in Table 1.

## Toxicity

Toxicities were reported according to the Common Terminology Criteria for Adverse Events (CTCAE) v. 5.0. At the end of pelvic EBRT, diarrhea CTC°I was reported in 3 patients (30.0%), proctitis CTC°I in 2 patients (20.0%), pollakisuria CTC°I in 2 patients (20.0%), dysuria CTC°I-II in 6 patients (60.0%), nycturia CTC°I-II in 5 patients (50.0%), urinary urgency CTC°I-II in 4 patients (40.0%), and dermatitis CTC°I-II in 2 patients (20.0%). No adverse events ≥ grade III were observed. There were no worsening of aforementioned acute toxicities after MRg-SBRT boost.

More than 3 months after MRg-SBRT boost, we observed CTC°I nycturia (40%), CTC°I dysuria (30%), and CTC°I urinary urgency (30%) as the most common side effects. Toxicities are summarized in Table 3.

**Table 3:** Acute toxicity during and 3 months after EBRT followed by MRg-SBRT boost, according to Common Terminology Criteria for Adverse Events (CTCAE) v. 5.0

Toxicity	Acute			≥ 3 months after SBRT		
	Grade I	Grade II	Grade ≥ III	Grade I	Grade II	Grade ≥ III
Diarrhea	3			1		
Proctitis	2			1		
Pollakisuria	2			1		
Dysuria	5	1		3		
Nycturia	5			4		
Urinary urgency	4			3		
Urinary incontinence		1		1		
Vaginal discharge	4			2		
Dermatitis	1	1		1		

## Discussion

In the present analysis we reported the early results of patients LARGC, who were ineligible for a sequential BT treatment and alternatively treated with an MRg-SBRT boost.

To date there are limited studies, which investigated the role of an EBRT boost in patients ineligible for BT. Barraclough et al. analyzed 44 patients with cervical cancer, who were not suitable for BT and therefore received a conventionally fractionated EBRT boost subsequently to pelvic irradiation. The EBRT boost was delivered with a total dose of 15.0–25.0 Gy in 8–10 fractions, resulting in a cumulative dose of 54.0–70.0 Gy. Even though late grade 3 toxicity was only seen in 2% of patients, recurrent disease was reported in 48% of the patients after a 2.3 year follow-up. The high recurrence rate was due to insufficient dose coverage in the large boost volume (median: 228 ccm) and dose limitations of the rectum, bladder and small bowel [16].

Other retrospective studies reported their experience on using SBRT to mimic BT in patients with LARGC, ineligible for BT. SBRT could be advantageous over conventionally fractionated EBRT, because of its ability to deliver higher dose to the tumor, and reduce the dose exposure of OAR at the same time. Guckenberger et al. evaluated the outcome of an SBRT boost after 50.0 Gy whole pelvic radiation in patients with LARGC. The SBRT boost was delivered with a total dose of 15.0 Gy in 3 fractions. There were no OAR dose exposure data regarding D0.1 ccm, D1ccm, and D2 ccm of the rectum. It was assumed the parts of the rectum receiving a higher dose in SBRT, were also fully exposed to EBRT of the pelvis. Despite the excellent local control rate of 81% after 3 years, grade ≥ 3 late sequelae were reported in 25%. Among 16 patients, 10.5% patients developed grade 4 intestinovaginal fistulae and 5.3% patients developed grade 4 small bowel ileus after a median follow-up of 22 months. Patients, who developed grade 4 intestino-vaginal fistulae, received a higher dose to the rectum (D_max_ 80–100 Gy EQD2_3_). Other factors that caused the high rate of toxicities were large target volumes (median PTV volume: 92 ccm), and the invasion of the pelvic wall in most of the patients [17].

Another more recent study from Albuquerque et al. analyzed 15 patients with locally advanced cervical cancer (LACC), who were unable to receive BT and were treated with a SBRT boost. A total dose of 28.0 Gy in 4 fractions was delivered to the PTV. The median PTV volume was 139 ccm, which was larger than the median PTV volume in the present study. The rectum received a median total dose of 90.6 Gy (D2ccm, EQD2_3_), which was much higher compared to our study. Similar to Guckenberger et al., they also reported 26.7% grade ≥ 3 toxicities, mostly rectal ulcers or rectovaginal fistulae related to patients with very large tumors. Two of these patients (13%) died from sepsis and bleeding after refusing colostomy [18]. Comparable rates of grade 2–3 toxicities were reported by Kubicek et al. despite a smaller boost volume [6].

Although the current National Comprehensive Cancer Network (NCCN) Guidelines for the treatment of cervical cancer do not recommend SBRT as an appropriate routine alternative to BT [19], SBRT boost might remain a salvage option in patients unable to receive BT. In the current study, a combination of pelvic EBRT and MRg-SBRT boost was well tolerated with encouraging early results. Due to superior soft tissue contrast, the use of MR-guidance allowed for better visualization of normal and tumor tissue compared to CBCT. Gynecological cancers are well known to have large inter-fraction movements due to different filling or surrounding organs [20]. The sequential MRIs during the SBRT boost enables to adjust not only the tumor and OAR volumes based on daily anatomy, but also to capture inter-/ intrafraction movement, and to re-optimize the treatment plan accordingly. Hence, we were able to maximize dose to the target volume, while sparing the OARs [21]. However, the authors would like to emphasize that MRg-SBRT should never replace the treatment of choice–which is and will remain brachytherapy.

Regarding the tumor volume, we reported smaller median PTVs than in the previous studies from Guckenberger et al. and Albuquerque et al. [17, 18], nonetheless the PTVs are comparable to other similar studies [6, 22]. In order to preserve the OARs and prevent high rates of toxicity, the median cumulative dose to the PTV was lower than the recommendation in the EMBRACE II protocol [14]. It has been reported in a previous study, that the D1ccm, as well as D2ccm of the rectal wall are predictive for chronic rectal toxicity, moreover applying traditional dose multimodal concepts of EBRT and BT resulted in acceptable late toxicities of 10–15% [23]. The results from the prospective multicenter EMBRACE study showed that D2ccm of the rectum ≤ 65.0 Gy was correlated with less frequent rectal morbidity [24]. Therefore, we followed all classical constraints from brachytherapy (EMBRACE II protocol) regarding rectum, bladder, sigmoid and bowel. More than 3 months after the MRg-SBRT, we found 30% grade 1 dysuria, 40% grade 1 nycturia, 30% urinary urgency, but no grade ≥ 2 toxicities.

With an early median follow-up of 9 months, we observed a local control rate of 90% with 6 patients with CR, 2 patients with PR, and 2 patients with PD. The patient with a locoregional progression also developed distant metastases 6 months after the therapy. She had the largest tumor volume among all patients (PTV = 131.35 ccm), with infiltration of the pelvic wall and other adjacent organs. This finding is in accordance with Ijaz et al., who reported extension to the pelvic wall as a negative prognostic factor for survival [25].

Some other retrospective studies analyzed the utilization of a SBRT boost in patients ineligible for BT. There were heterogeneous results in terms of toxicity and outcome. Unknown tumor volume, different radiation techniques and fractionation schedules make it difficult to compare them to our results [5, 6, 17, 18, 22, 26–29]. A summary of the current literature is described in Table 4.

**Table 4:** Summary of literatures on SBRT boost in patients ineligible for BT

Study	Number of patients and diagnosis	Total dose of SBRT boost/number of fractions (Gy/fx)	Median PTV (ccm)	Median follow up (months)	Toxicity	*Outcome after* ≥ *3 months after therapy*
Albuquerque et al. (2019)	15 LACC	28/4	139	n/a	26.7% G3 rectal ulcer/fistula	2-yr LC 70.1% 2-yr PFS 46.7% 2-yr OS 53.3%
Kubicek et al. (2013)	3 recurrent cervical cancer; 4 LACC; 2 endometrial cancer; 2 vaginal cancer	25/5	9.2	14	18% acute G2 dysuria and GI toxicity 9% G3 late GI-toxicity (rectal bleeding)	3 patients died (2 with recurrent cervical cancer, 1 patient with LACC) 1 patient had local recurrence 3.5 years after SBRT
Haas et al. (2012)	6 LACC	20/4 19.5/3	n/a	14	No toxicities	No local recurrence
Molla et al. (2005)	9 endometrial cancer 7 cervical cancer	14/2 20/5	n/a	12.6	No ≥ G3 acute toxicities Late toxicities: 6% G3 GI-toxicities (rectal bleeding) 6% G1 abdominal pain 38% G1 sexual toxicity (vaginal dryness, synechiae, dyspareunia)	6% recurrence after 12.6 months
Guckenberger et al. (2010)	16 recurrent cervical cancer and endometrial cancer	15/3	92	22	10.5% G4 intestinovaginal fistulae 5.3% G4 small bowel ileus	3-yr OS 34% 3-yr LC 81%
Kemmerer et al. (2013)	11 unresectable endometrial cancer (stage I-III)	30/5	n/a	10	No late toxicity	1-yr FFP 68%
Higginson et al. (2011)	5 (1 endometrial cancer, 2 vaginal cancer, 1 cervical cancer, 1 urothelial carcinoma)	20/5 25/5 16/4	n/a	11	20% G3 rectal bleeding	4 patients died. 1 patient had no evidence of disease
Marnitz et al**.** (2013)	11 LACC	30/5	48.9	6	No G3 GU and GI acute toxicity	No local recurrences after 6 months
Ito et al. (2019)	6 LACC	19.5/3 21/3 22.5/3	n/a	17	No G3 toxicities	100% LC at last follow-up 1 distant metastasis at last follow-up
Our study (2021)	5 recurrent cervical cancer 2 LACC 3 recurrent vaginal cancer	21.0/4	43.5	9	No G3 toxicities	6 CR 2 PR 2 PD

*LACC* locally advanced cervical cancer, *LC* local control, *PFS* progression-free survival, *OS* overall survival, *CR* complete response, *PR* partial response, *PD* progress disease, *FFP* freedom from progression, *GI* gastrointestinal, *GU* genitourinary

The current study inherits several limitations. A longer follow-up is necessary, to rule out high grade chronic toxicity and to obtain long-term outcomes. Our cohort was small and heterogeneous, so that a comparison between the efficacy of a SBRT boost and BT is difficult. However, as high-dose levels to the HR-CTV are very important for local control rates [30], BT will always remain superior and the standard of care [21]. Therefore, MRg-SBRT boost may serve as a backup solution in selected patients, who are ineligible for BT. The current study revealed that MRg-SBRT seems a safe therapeutic option and could be favorable over CBCT-guided SBRT.

## Conclusion

These early results report the feasibility of an MRg-SBRT boost approach in patients with LARGC who were not candidates for BT. When classical BT-OAR constraints are followed, the therapy was well tolerated. However, long-term follow-up is needed to validate the present hypothesis.

## Data Availability

The datasets used and analyzed during the current study are available from the corresponding author on reasonable request. Research data are stored in an institutional repository and will be shared upon request to the corresponding author.
